# Breaching Trust: A Qualitative Study of Healthcare Experiences of People Who Use Drugs in a Rural Setting

**DOI:** 10.3389/fsoc.2020.593925

**Published:** 2020-11-10

**Authors:** Kaitlin Ellis, Suzan Walters, Samuel R. Friedman, Lawrence J. Ouellet, Jerel Ezell, Kris Rosentel, Mai T. Pho

**Affiliations:** ^1^Pritzker School of Medicine, University of Chicago, Chicago, IL, United States; ^2^Rory Meyers College of Nursing, New York University, New York, NY, United States; ^3^Department of Population Health, NYU Grossman School of Medicine, New York, NY, United States; ^4^COIP/Epidemiology and Biostatistics, University of Illinois Chicago School of Public Health, Chicago, IL, United States; ^5^Africana Studies and Research Center, Cornell University, Ithaca, NY, United States; ^6^Section of Infectious Disease, Department of Medicine, University of Chicago Medical Center, Chicago, IL, United States

**Keywords:** rural, opioid, inject, drugs, healthcare, stigma, access, barriers

## Abstract

**Background:** Increased drug use has disproportionately impacted rural areas across the U.S. People who use drugs are at risk of overdose and other medical complications, including infectious diseases. Understanding barriers to healthcare access for this often stigmatized population is key to reducing morbidity and mortality, particularly in rural settings where resources may be limited.

**Methods:** We conducted 20 semi-structured interviews with people who use drugs, including 17 who inject drugs, in rural southern Illinois between June 2018 and February 2019. Interviews were analyzed using a modified grounded theory approach where themes are coded and organized as they emerge from the data.

**Results:** Participants reported breaches of trust by healthcare providers, often involving law enforcement and Emergency Medical Services, that dissuaded them from accessing medical care. Participants described experiences of mistreatment in emergency departments, with one account of forced catheterization. They further recounted disclosures of protected health information by healthcare providers, including communicating drug test results to law enforcement and sharing details of counseling sessions with community members without consent. Participants also described a hesitancy common among people who use drugs to call emergency medical services for an overdose due to fear of arrest.

**Conclusion:** Breaches of trust by healthcare providers in rural communities discouraged people who use drugs from accessing medical care until absolutely necessary, if at all. These experiences may worsen healthcare outcomes and further stigmatize this marginalized community. Structural changes including reforming and clarifying law enforcement's role in Emergency Departments as well as instituting diversion policies during arrests may help rebuild trust in these communities. Other possible areas for intervention include stigma training and harm reduction education for emergency medicine providers, as well as developing and implementing referral systems between Emergency Departments and local harm reduction providers and medically assisted drug treatment programs.

## Introduction

People who use drugs, especially via injection, are at increased risk for medical complications, including HIV, hepatitis C virus (HCV), sexually transmitted infections (STIs), endocarditis, skin and soft tissue infections, psychiatric illnesses, and overdose (O'Connor et al., 2014; Kievlan et al., 2015; CDC, 2018, 2019). It is also highly likely that people who use drugs are at increased medical risks from COVID-19 (Vasylyeva et al., 2020; Volkow, 2020). Despite these risks, people who use drugs access primary care less often and utilize emergency medical services (EMS) more frequently than the general population. The under-utilization of preventative healthcare services in this population can lead to a variety of adverse health outcomes, including high infectious disease prevalence and poorer mental health, as well as higher costs from medical complications and overuse of EMS (French et al., 2000; Ahern et al., 2007; Patrick et al., 2012; Artenie et al., 2015; Paquette et al., 2018; von Hippel et al., 2018).

One explanation for the under-utilization of medical care by people who use drugs may be their experiences with stigmatization. (Earnshaw and Chaudoir, 2009) conceptualized the theory of enacted and anticipated stigma of those living with HIV and have since adapted the concept to people who use drugs (Earnshaw et al., 2013). The authors define enacted stigma as “experiences of prejudice, stereotypes, and discrimination from others in the past” and anticipated stigma as “expectations of prejudice, stereotypes, and discrimination from others in the future” (Earnshaw et al., 2013, page 3). The enacted stigma that people who use drugs face from society, as well as the associated adverse effects on risk behavior and health outcomes, is well-documented. For example, research by Friedman et al. (2017) showed that after people who inject drugs experienced interpersonal attacks on their dignity, they partook in riskier health behaviors, such as needle sharing. Similarly, an Australian study found that among people who inject drugs, those who reported discrimination in the past 12 months had elevated rates of overdose, diminished physical functioning, and mental illness (Couto e Cruz et al., 2018). The primary sources of discrimination reported by participants in this study included pharmacies, doctors, and hospitals. This correlates with findings that medical providers may harbor negative attitudes toward people who use drugs and often lack sufficient training or knowledge to address their medical concerns (Merrill et al., 2002; Brener et al., 2010; van Boekel et al., 2013; Pullen and Oser, 2014; Chiarello, 2016).

Rural communities are disproportionately burdened by opioid and methamphetamine use in the United States (Ellis et al., 2018; Palombi et al., 2018; Baker et al., 2020). Stigma and other barriers to accessing medical care can be magnified in rural settings where there are fewer options for healthcare services and perhaps less anonymity in medical interactions (Jones et al., 2009; Pullen and Oser, 2014; Buer, 2020). This problem is compounded by a fear of legal consequences that can follow the identification of illicit drug use by a patient, such as incarceration or losing custody of their children (Koester et al., 2017; Latimore and Bergstein, 2017). Such legal consequences and vulnerability to discrimination and stigmatization may be exacerbated by recent changes to rules governing confidentiality of patient records in substance use disorder treatment (Knopf, 2020). Despite the profound impact that such barriers have on health outcomes, few studies have elicited the experiences and perceptions of people who use drugs regarding healthcare interactions, particularly in rural settings. Understanding the healthcare experiences of people who use drugs in rural communities can help inform interventions that improve access to, and quality of, healthcare for this vulnerable population.

Through qualitative interviews in rural southern Illinois, we explored people who use drugs' experiences with healthcare systems, particularly Emergency Departments (ED) and EMS. In this paper, we describe the barriers they faced when seeking medical care and their responses to those barriers. We also explore the role law enforcement played in their medical decisions and experiences.

## Materials and Methods

This study reports findings from qualitative data of the Delta Rural Health Study, a member of the multisite Rural Opioid Initiative (ROI) cooperative agreement (see Funding). The ROI focuses on understanding rural opioid use and the potential for HIV, HCV, and other sexually transmitted infections in nine rural regions of the United States.

### Study Setting

The study was performed in the Illinois counties of the Delta Regional Authority, an understudied area with high rates of HCV infection and drug overdose (Illinois Department of Public Health, 2017). This region consists of the 16 southernmost counties of Illinois: Randolph, Perry, Franklin, Hamilton, White, Jackson, Williamson, Saline, Gallatin, Union, Johnson, Pope, Hardin, Alexander, Pulaski, and Massac. According to the 2018 American Community Survey, these counties are predominantly rural, with an average population of 20,623 and the region has substantially lower median household incomes than Illinois as a whole (United States Census Bureau, 2019).

### Participant Eligibility and Recruitment

Participants had to be at least 15 years old, report injecting any non-prescribed drug or using non-prescribed opioids by any route in the past 30 days, reside in one of the aforementioned counties, speak English, and provide informed consent. Participants were recruited from three sources: (a) an indigenous local harm reduction organization (HRO) that provided mobile syringe services, naloxone and HIV/HCV/STI testing, (b) persons who completed the study's survey component and referred additional participants as part of an incentivized respondent-driven sampling approach (Heckathorn, 2011), and (c) a community-based drop-in center that primarily served people experiencing housing instability. As interview recruitment progressed, participants were purposively selected to provide variation in demographics, drug of choice, and county of residence, as well as variations in experiences with medical care among persons who knew themselves to be HCV-positive.

### Data Collection

All participants completed the informed consent process. Persons incapable of informed consent due to drug withdrawal or intoxication were rescheduled. Three members of the research team conducted semi-structured, audio-recorded narrative interviews with participants using an interview guide described below. Interviewers had no affiliation with healthcare providers used by people who use drugs in the study area. Interviews took ~45–90 min and were audio-recorded. A unique ID and pseudonym were created for each participant, and interview transcripts were de-identified. Participants were paid $40 cash for their contribution. The study protocol was reviewed and approved by the institutional review board at the University of Chicago and participant involvement was covered by a Federal Certificate of Confidentiality.

### Interview Guide

The interview guide was collaboratively developed by researchers from all sites participating in the Rural Opioid Initiative and aimed to investigate sociocultural factors associated with illicit opioid or other drug use, high-risk drug and sex behaviors, harm reduction, and social network characteristics. It also explored factors impacting healthcare and social service utilization, treatment for substance use, experiences with law enforcement, and knowledge of laws regarding drug paraphernalia, naloxone (an opioid reversal agent), and overdose reporting. Specific questions regarding healthcare experiences included “Have you ever decided that you needed care, but didn't go?” and “Tell me about your most recent interaction with any doctor or other health care provider.” Demographic data were collected with each interview.

### Data Analysis

Recorded interviews were professionally transcribed and then reviewed by interviewers to correct transcription errors and omissions. After finishing an interview, interviewers made notes regarding the interview, including identifying potential themes. Data analysis combined structural coding (Guest et al., 2012) reflecting specific topics of interest at the study's inception and a modified grounded theory approach where themes are coded and organized as they emerge from the data (Charmaz, 2006). A primary coder developed a code book of mostly *a priori* codes based on the interview guide and then coded one transcript, refining the code book throughout the process. A second coder coded the same transcript to check for fidelity and overall consistency in the application of codes. Once these codes were agreed upon, iterative coding was conducted by the primary coder for the remaining 19 transcripts. For this study, further thematic analysis was conducted regarding participants' experiences with medical care providers, with a focus on barriers to seeking or accessing medical care and participants' responses to these barriers. Transcript coding and analysis was done in NVivo 12^Ⓡ^.

## Results

Twenty participants were interviewed, their mean age was 36.6 years and the majority were white (90%), which aligns with census data for this area, and male (65%). In the 30 days before their interview, the average number of drugs used was 4.6 and over half (17) of participants had used methamphetamine. Other commonly used drugs were prescription anxiety drugs (12 participants), heroin (10 participants) opioid painkillers (10 participants) and cocaine or crack (10 participants). There are no definitive descriptions of drug use prevalence in the area studied, but our sample appears to reasonably align with substance use patterns suggested by local harm reduction providers, drug treatment programs, police drug seizures, and newspaper reports.

The primary finding is that participants reported multiple, interrelated barriers to seeking medical care. Structural, financial, and interpersonal issues often led participants not to seek care or created barriers when they sought care. Structural and financial barriers included limited nearby services, lack of transportation, and inability to pay for care. However, interpersonal factors, many of which were linked to participants' prior experiences with medical services, were described by participants as the most formidable barriers to care.

We divided interpersonal factors into three broad themes: (1) stigma, (2) inappropriate treatment, and (3) fear of negative consequences. Participants' responses to these barriers were included within each theme. We found that most of the medical services discussed were provided by EMS or EDs and their associated providers and that law enforcement interactions played an important role in participants' healthcare experiences and decisions. We use pseudonyms throughout the paper when quoting participants.

(1) Stigma

Descriptions of stigma and discrimination were pervasive throughout participants' narratives. Enacted, or past experiences of stigma, led to expectations of future stigma, which affected participants' healthcare seeking decisions.

(a) Enacted Stigma:

Participants reported being treated poorly or differently by medical providers as a result of their drug use or providers' assumptions about their drug use. Participants recalled being identified as a person who used drugs by drug testing, track marks on their arms, or the inability of staff to access veins for blood draws. After being identified as a person that used drugs, participants felt immediately stigmatized and mistreated. Sam, a 40-year-old man, described a medical interaction as “[…] horrible, horrible. They treated me like shit because they knew I was a drug user.” He went on to describe what he experienced as a forced catherization.

It was a local hospital up here in [Town A]. They treated me like shit. They obviously knew that… I had scarred veins and stuff like that… They needed me to pee for urinalysis just to see what was in my system and they said that if I couldn't… I told them I didn't have to pee but if they brought me some water that I'd be able to drink the water and give me about 15 minutes and I'd be able to pee. They brought in the water cup and they let me take one sip of it and then they asked me to pee. I told them I couldn't and they catheterized me… They held me down and cathed me, yeah… It wasn't the best experience.

Maya, a 60-year-old woman, Walter, a 33-year-old man and Sarah, a 38-year-old woman, respectively, described being treated like a “second class citizen,” “piece of crap,” and “drug addict” in medical settings. Maya explicitly stated “I don't like going to the ER because you're treated like a junkie.” Emily, a 27-year-old woman, described an initial evaluation that she felt led to stigmatizing treatment.

When I have had to go to the ER for anything, the first thing they do is drug test. Like if you go in for a legit reason, like something's really wrong, and the first thing they do is drug testing, like, “Well, you're not going to treat somebody that's on drugs?” They make it a point to have you drug tested and then they want to. You get treated different if you do fail your drug test in there.

These examples show how participants felt stigmatized, dehumanized, and mistreated by medical providers based on their identification as people who use drugs when they accessed care. The next section explores how past experiences of discrimination affected decisions about future utilization of healthcare.

(b) Anticipated Stigma:

Participants often described situations in which anticipated stigma, in the form of judgment or discrimination, discouraged them from accessing medical care or disclosing their drug use to providers. Rob, a 42-year-old man, described being “too embarrassed” to seek care for an injection-related abscess. Similarly, Jack, a 43-year-old man, expressed a concern about facing stigma due to a medical diagnosis when asked if anything kept him from seeking care.

Yeah. Yeah. Because pretty much if you got hep C nowadays, it's because you were an addict. That's usually pretty much the only way nowadays that people have it. They're going to know you're an addict…I don't want to be judged.

Due to anticipation of stigma or mistreatment, participants often reported avoiding medical care. Alex, a 40-year-old man, said he would not seek care unless he was “in extreme pain,” and Emily explained “I don't ever see any doctors or I try to avoid the ER at all possible costs.” When asked about one healthcare setting, Walter recalled being “treated fine there, so long as it's not for drugs” but when pressed further about his experiences he responded “I try not to get sick much.”

Some participants sought care but described strategically withholding disclosure of their drug use to providers to avoid judgement. As Kelly, a 30-year-old woman, responded when asked if she discloses her drug use: “No, I don't. Not until it comes down to it. Especially if. Because I don't want everybody that comes across my paperwork to read it.” A few reported that they generally were upfront about using drugs. For example, Anthony, a 25-year-old white man, initially told us that he was “totally comfortable” making this disclosure, although he described providers' reactions as “disbelief” due to his “clean-cut” appearance. This description suggests that white race and a middle-class appearance can be deployed to offset at least some of the stigmatization that a person using drugs is likely to encounter when seeking medical services. However, in section 2, below, Anthony also described using a calculus to decide when to disclose.

Jack described the relationship between needing pain medication for an injury, but also wanting it for his “addictive mentality” and struggling with how much to tell his providers in order to be treated like a patient and not an addict.

So, I need to go get stitches. Me not wanting to look like an addict, but at the same time I knew I was in pain then and I knew I'd be in pain after they'd put the stitches in, so I wanted pain pills, and my addictive mentality wanted the pills too. Over the years, I have been looked at, looked down on because I was an addict and treated different in a hospital. I don't think that's right. Your job is there to treat the problem but you had so many addicts going in there and trying to work the system to get free pain pills… That makes it look bad for the people that actually kind of need them… Myself included, I've done it myself. Went and said I had a backache or a toothache and nothing was on me just so I can get pain pills… But then when you need them, you don't want to ask because then if you already know what you're talking about and what you're asking for, they'd pretty much know you're an addict, that's some con… They do treat you different.

Overall, between enacted stigmatizing behaviors by medical providers, and the anticipated stigma that participants felt they would face in medical settings, stigma played a key role in participants' medical decisions and acted as a barrier to seeking care and disclosing important health information.

(2) Inappropriate Treatment

Participants described experiences in which they felt their medical issues were undertreated or inappropriately treated because of their drug use, particularly regarding pain management.

Participants described not receiving the medical attention they needed and providers not taking their pain or medical concerns seriously. Sometimes participants felt they were undertreated because providers assumed that they were seeking drugs. In response, Anthony said that the decision to disclose his drug use depended on the reason he was seeking medical care: “Yeah, so I don't know, it just depends on what the reason that I'm going. If I'm going because I have respiratory issues going on, I'm going to tell them the truth. If I go there because I broke a rib, I'm going to lie.”

Other participants attributed their poor care and follow up to providers' perceptions of them as drug users and their past experiences of stigmatization in medical settings. For example, Matthew, a 27-year–old man, described long wait times and frustrating results from his local emergency department.

Like I said, you don't get, you can't go in off the street and get real care there… You can't, it's very, very hard to go to that ER without being there for two or three hours and walking out with nothing other than more resentment, more frustration and pain and anxiety and feeling more wronged and more dehumanized and less trust and faith in your own nation. Honestly, every day of my life, I'd give anything just to have real healthcare and real support health wise.

Kelly described experiences of visiting multiple hospitals in unsuccessful attempts to receive adequate care.

Oh, I mean just in general. We call [facility A] the band aid hospital. Sometimes they give us our kind of band aid and push them on their way. Other hospitals give our… We end up leaving that hospital and go straight to another one because they're still bleeding, and they give them a suture or two, or something like that.

While some participants described having sought pain medication from EDs or other providers when they were not in need of medical treatment, they also described situations in which they were in considerable pain from legitimate medical conditions but felt they were not treated appropriately because of their drug use history. For example, Maya explained, “you go to the hospital and they won't so much as give you a pain shot because they see track marks and they think you're fishing for pain medicine, when you're in legitimate pain.”

Other participants described specific situations in which they believed their medical issues had been undertreated. Trevor, a 38-year-old man, described being left “on the back burner” at an ED when he tried to receive care for a neck abscess. He also stated that ED staff were “profiling” to determine who to give pain medication to: “If you're older and you look straighter, if you look fine, good cleaned up and look fine, they'll give you [pain medication].” In another example involving mental health, Allison, a 38-year-old woman, described self-medicating in response to the long wait times to see a psychiatrist: “Here, you can't get into one for months at a time and people have got to get rid of that, that whatever, anger, hurt, and dulling it is the easiest way to do it. It's easy to do with drugs.” David, a 39-year-old man, spoke of the risk of being denied medical care all together because of drug use: “they might not treat you after they find out you're on drugs.” Overall, as Evan, a 57-year-old man explained about his local hospital, a general sentiment was that hospitals “ain't doing shit” for the medical concerns of people who use drugs.

There were a few examples in which participants perceived they had been inappropriately treated with psychoactive substances by medical providers. Megan, a 38-year-old woman, expressed that she felt a provider had overprescribed her anti-anxiety medication that she did not need at the time. Kelly noted cutting the dosage of her opioid prescriptions in half because her providers gave her dosages that were too high. She went on to describe medical providers as “Pez dispensers.” In another example, David, who primarily injected methamphetamine, described convincing a nurse to inject him with his prescribed pain medication in a rehabilitation facility because it did not work fast enough when he took it orally.

And so they started with the therapy really intense, and it hurt. It hurt so bad, and just taking the pills just wasn't fast enough.I talked one of the nurses into breaking one down for me. And she injected it for me for the first time. And it worked. So she started breaking them down for me, and I started injecting myself. After 6 weeks of doing this, they released me from the hospital, and they put me through pain therapy, and they took me off of it, and it was hell. Started looking for them on the street.

A common result of these negative experiences as expressed by participants was to lose faith and trust in their medical system. As Anthony described about providers, “They cover each other's asses. I don't like it. They're not… I just don't trust them, and for good reason.” Matthew described his feelings that medical providers are more worried about their livelihoods than their patients and that medical institutions' financial concerns are more important to them than patient care.

They don't care about people at all. They care about the student loans they had to take out to go to school to work at that hospital taking care of other people. I see it every day and it just breaks my heart.

When participants felt their medical concerns were ignored or they were inappropriately treated, they chose to hide their drug use from providers, treat themselves, or give up on the system and avoid accessing care all together.

(3) Fear of Negative Consequences

Another barrier to accessing medical care was a fear of negative legal and social consequences. These consequences came in two general forms, the most common was a fear of law enforcement involvement due to participants' drug use. This fear was often cited when EMS responded to an overdose. The other form was a fear of breaches in participants' confidentiality, which could harm their reputation in their communities. We found that these fears were often rooted in both past personal experiences and the experiences of others known to participants.

(a) Fear of law enforcement involvement in medical interactions:

Participants described fears of encountering law enforcement and possible arrest when seeking medical care while they had drugs in their system or were carrying drugs. Ryan, a 35-year-old man, explained that he never goes to the doctor when he has drugs in his system because “(I) don't want to be investigated.” Trevor described overdosing and begging not to be taken to the hospital because “I had meth on me.” When asked about how she decides when to access care and how she is treated in local ERs, Emily stated she avoids the ER “at all possible costs” and reported a time that her brother sought medical attention and was arrested for drug possession in the ED.

My brother actually went to the ER. I don't even remember what he went for. And they drug tested him and they called the cops on him. And they're not even supposed to do that. And he ended up going to jail because they called the cops because he failed his drug test and they didn't like him anyways… But, they called the cops on him and then the cops come up there and searched him at the hospital. And he had drugs on him, so he went to jail.

Heather, a 31-year-old woman, was convinced that if she sought medical care with drugs in her system, her medical test results would be shared with law enforcement

I haven't been to the doctor in a long time. When I'm on meth, I don't go to the doctor. You go. if you go to the doctor on meth, and they drug test you, then they fucking file a report with the police, like every fucking time.

Sarah reported not wanting to be treated by a specific ED provider who had previously let police officers into the area where she was being treated because the provider believed she was lying about the causes of her injuries.

In another form of medical service and law enforcement interaction, Maya and Kelly described experiences in which police officers took their legal medications or legal prescription slips from their homes or cars during searches. In both cases, the participants' access to legally prescribed medications was delayed. These actions were seen as disrespectful and dehumanizing, as they suggested that law enforcement concerns outweighed medical care.

Based on personal or second-hand experiences, participants' felt that law enforcement was frequently inappropriately included in their medical care and that accessing medical care, especially while using drugs, would lead to their arrest or investigation.

(i) Fear of calling EMS for an overdose:

Most fears of law enforcement involvement in medical interactions regarded accessing EMS in response to an overdose. While many participants described themselves as willing to call 911 despite experiences or fear of arrest, they recounted others' unwillingness for the same reasons due to, as Anthony put it, a “culture of fear.” Multiple participants recalled situations in which EMS was not called when a person was overdosing. The decision to avoid calling 911 was almost always attributed to fear of legal repercussions. Kelly describes one of these instances:

His whole body was swelling up, his feet, his arms, everything. I told everybody ‘I think he's overdosing.' But nobody had a car to take him to 911. The other people I was with didn't want 911 called because they were at a house that had a bunch of drugs at it.

When EMS was called for an overdose or any other reason, police officers were said to be the first to respond. Participants felt that in these situations law enforcement was often more concerned with arresting people or finding evidence for arrests than with addressing the emergency that prompted the call. For example, Emily described being arrested due to an outstanding warrant for a missed court appearance when she called 911 for a friend who had overdosed. Maya described a situation in which police officers first searched her house instead of administering CPR or naloxone while she was overdosing.

They've been called here numerous times that I've overdosed. Cops have to show up before the ambulance. One time, I was sitting in that chair and I was turning bluer and bluer and [husband] called 911. They came in and they said, ‘Well you're going down for homicide,' to [husband]. He says, ‘I don't care what the fuck you arrest me for. Get her to a hospital. She's turning bluer.'…They sat there and they started searching through the house, dumping out the garbage can in the bathroom and he said, ‘Hey, I didn't give you permission to go through my house. I called 911 to take my wife to the hospital.' …But I sat there for 20 minutes while they argued with [husband], turning bluer and bluer.

Some participants expressed negative attitudes toward EMS, either because they associated them with law enforcement, or felt they behaved as law enforcement rather than medical providers. Sam described not calling EMS when a friend was overdosing because “I just don't like law enforcement at all.” Matthew explained, “EMTs, people who drive and operate ambulances and go to the scenes of crashes and crimes, they need better bedside manner. Every day people are suicidal and having panic attacks and anxiety attacks. When the ER shows up and they act like fucking cops. The cops in this town have more of a bedside manner than the ambulance people.”

Despite these negative experiences, Anthony believed the police had been treating people better since they had been trained to deliver naloxone to those overdosing: “They're trained more to worry about saving this person's life instead of worrying about what fucking…Ted or Alan's got in his fucking cabinets.” However, Anthony also believed a police officer administered CPR improperly to an overdose victim because “you don't want to get a little fucking junkie fucking saliva on your mouth?” Another participant, Walter, described waking up from an overdose in jail, rather than in a hospital: “they had to hit me like six times with [naloxone] and found out I had a warrant so (I) came to in jail.”

Overall, fear of law enforcement involvement, leading to investigation or arrest, was a factor in many of participants' decisions around medical care, and often acted a direct barrier to accessing care, particularly when calling EMS for an overdose.

(b) Fear of breaches in confidentiality:

Another feared consequence that served as a barrier to accessing medical care was that private medical information would be inappropriately shared by medical providers. This was especially relevant given the rurality of the area and the small-town nature of the communities described throughout participants' narratives. For example, when asked if he is worried about a local provider performing his STI testing, Evan expressed concern that his test results may be shared by the local health department. His response suggested that he is skeptical about the confidentiality of his test results when local health departments communicate with each other about disease outbreaks.

Yeah, they rap a lot. It's like, let's say, [Town C], [County A] get a big case of AIDS, like how they know that? A person that may have it they don't go advertising it. So that's some people talking. So, you be skeptical about going to these places around here.

Two participants, Rob and Emily, described experiences in which their confidentiality was breached by medical providers. The first involved a receptionist who Rob believed disclosed his HIV status to the entire medical staff “so when I walked out they all turned around, looked…” This experience caused Rob to feel “very hesitant of who I share my status with when it comes to medical offices because of that situation.” The other breach came in the form of a provider disclosing the content of Emily's counseling sessions to community members.

I try to avoid conversation like that, because I was seeing a counselor in [clinic name] a couple of years ago, and was talking to them about everything going on in my head and the pills… and all that stuff was supposed to be confidential, and she actually told. she said something about it to numerous people. People were coming to me saying, “Is this your counselor at [clinic name]?” I was like, “Yeah.” And they said, “Well, she's telling people everything you say in there.” And she even went to my mom with it too. She told my mom certain things… Yeah. So I tried. that's something that I really try to avoid talking to people about. That's really the last thing I need to get out. And I think that that's why I've not really went to rehab or tried to go to rehab to get help.

Emily explained that she chooses not to disclose her drug use now to providers because of that experience. These two experiences describe how past negative medical experiences directly affected participants' future decisions regarding seeking care and disclosing important health information to providers.

## Discussion

Trust in a provider is vital to the health and well-being of the patient. Trust allows for a shared decision-making process regarding medical care, which has been shown to improve health outcomes (Peek et al., 2016). In our analysis, we found that participants felt that providers often breached this trust through stigmatization, mistreatment, involvement of law enforcement, and violations of confidentiality. We also found that the blurred relationship between the criminal justice and healthcare systems in these communities fostered mistrust in the intentions of medical service providers and created, as one participant described a “culture of fear” that affected participants' healthcare decisions. Our participants made it clear that these breaches in trust discouraged them from accessing medical care and, when they did access care, from disclosing their drug use, associated risk behaviors, and even previous medical diagnoses to medical providers.

Our study adds to the limited body of research that qualitatively explores the experiences of people who use drugs with healthcare services. Previous works have studied different healthcare service types, generally have not interviewed people who actively use drugs or were conducted in large urban centers (Earnshaw et al., 2013; McKnight et al., 2017; Paquette et al., 2018; Biancarelli et al., 2019). Our study contributes to the literature by investigating the experiences of people who actively use drugs when navigating multiple rural healthcare settings including EDs and EMS, but also with mental health, infectious disease and primary care providers.

Although many of our findings reinforce the current literature on the stigma people who use drugs experience in healthcare settings, we also found concerning new themes that warrant further investigation. Our study reinforces previous work that shows people who use drugs are fearful of utilizing EMS for an overdose due to the possibility of arrest (Koester et al., 2017; Latimore and Bergstein, 2017). This finding is important as many of our participants' healthcare interactions began with a call to EMS, to which law enforcement was often the first responder. Wagner et al. (2019) similarly found that people in urban areas who use drugs equated a 911 call for a medical reason to calling the police. Our study found that this sentiment may be exacerbated in rural settings where our participants were often well-known to a small police force and, not uncommonly, had ties to some officers through shared school histories, neighborhoods or family relations.

Our study also shows how this fear extends to EDs and other healthcare settings, with greater consequent negative impact on healthcare decision making than previously reported. This fear is likely to be intensified by recent regulatory changes to the way patients' substance use disorder treatment information is stored and shared (Knopf, 2020). These new rules allow opioid treatment programs to input patient information into Prescription Drug Monitoring Programs (PDMPs), which law enforcement has the potential to search, as well as expand the circumstances under which patient information may be shared with law enforcement. The concern is that these new rules will discourage patients from seeking opioid use treatment in order to avoid persecution and discrimination. Studies have shown that law enforcement interactions increase rather than decrease health risk behaviors such as injection initiation and syringe sharing (Melo et al., 2018; Park et al., 2019) and that law enforcement involvement in the life of a person who uses drugs is almost unavoidable in the U.S. (Winkelman et al., 2018; Green et al., 2019). Therefore, a closer look at the way these interactions occur in rural medical settings and for medical purposes is critical to ensure that the rights of people who use drugs are upheld and their health is prioritized.

Our examination of the experiences of people who use drugs in rural southern Illinois uncovered a broad array of problems, from stigmatizing attitudes, loss of privacy, and poor care to active harm at the hands of their caregivers. While all served to diminish patient trust and confidence in the healthcare system, these diverse experiences reflected a multitude of failures along the cascade of providing a therapeutic interaction for people who use drugs. We suggest several potential interventions to address this range of issues:

First, reinforcing basic principles of confidentiality in patient-provider interactions within healthcare settings, especially as they apply to law enforcement, could begin the process of rebuilding trust between people who use drugs and the healthcare system. This exercise will be difficult, as law enforcement's rights in healthcare settings are often ambiguous and vary across the country (Jacoby et al., 2018). As a result, the American College of Emergency Physicians, along with researchers, have called for clear, universal guidelines, and policies for EDs, which could be developed with the input of community members (Tahouni et al., 2015; American College of Emergency Physicians, 2017). If implemented, policies could be communicated using signage and language in clinic and hospital handouts and posters clarifying protocols regarding protected health information (PHI) and law enforcement involvement. The signage could also convey positive messaging for people who use drugs, encouraging their access of healthcar,e and use of harm reduction services and practices.

Second, trainings on common diseases and complications faced by people who use drugs and the important public health consequences of these conditions may offer healthcare providers the tools, knowledge, and motivation to focus on best-practice treatments for this population rather than react to their stigmatized behaviors and appearances. These trainings can include direct stigma training, which has been shown to reduce stigmatizing attitudes by healthcare providers in the care of people living with HIV and may be equally efficacious in changing attitudes toward people who use drugs (Stringer et al., 2016). The use of trauma informed care (TIC) should also be incorporated into these trainings, which emphasizes understanding and responding to behavior through the lens of trauma, as well as cultural sensitivity and focusing on patients' safety and control in their medical interactions (Bassuk et al., 2017). TIC has been recommended in the treatment of all marginalized communities, and would be especially important in people with substance use disorders who have been shown to experience high rates of trauma (Konkolý Thege et al., 2017).

Third, the development of referral systems, especially out of EDs, to link people who use drugs to drug treatment and harm reduction services is another important step. Referrals that enable same day intake and treatment initiation, i.e. warm-hand-offs, are especially likely to improve patient follow-up and outcomes (D'Onofrio et al., 2015, 2018; Ahmed et al., 2019; Kelly et al., 2020). Recent work has also found success in incorporating telehealth and text messaging into referral systems to help with patient follow up, which may be especially useful in rural settings (Kmiec and Suffoletto, 2019; Wootton et al., 2019). Such approaches could be a major step in fostering therapeutic alliances between providers and people who use drugs. Lastly, an important step is expanding harm reduction services for persons not ready or able to enter drug treatment, especially syringe exchange programs, which have been associated with less stigmatizing care and reductions in infectious disease rates (Bluthenthal et al., 2000; Huo and Ouellet, 2007; Walters et al., 2017). Giving providers the resources to properly address the medical concerns of people who use drugs could facilitate more positive, open, and productive relationships with these patients.

In regard to the criminal justice system, many interventions are being developed across the country that, if implemented in these communities, may help improve trust in law enforcement among people who use drugs. One potential strategy used in Massachusetts includes post-overdose outreach by police officers and firefighters who helped connect persons who overdosed to care and support (Koh et al., 2018). The Law Enforcement Assisted Diversion (LEAD) and Angel programs, which have already been adopted by 20 sites and 28 states, respectively, are proven to reduce drug arrest charges in participating departments (Koh et al., 2018). These are two of many ways systems in rural communities can change to prioritize health over arrest for people who use drugs and, in turn, improve community health. However, the particular nature of these interactions in rural areas must be considered when adopting any new policy or process. Other research suggests, and our findings confirm, that people who use drugs may be unwilling to call 911 in the first place, especially if they know and have had negative interactions with local officers. Interventions in rural communities may benefit, therefore, from developing a separate contact protocol for any diversion or referral programs, rather than initiating the process through a 911 call or visit to police department, like some current models. Alternatively, a 911 call for an overdose could initiate both a police response and an advocate response, in which trusted harm reduction personnel are simultaneously called to the scene and can facilitate communication and diversion. Finally, an option may be to limit the scope of police when responding to drug overdose calls, prohibiting them from searching for drugs and narrowing the conditions for which people could be arrested at the scene, such as violent felonies. Ultimately, with or without the adoption of diversion programs, our data makes it clear that the current EMS protocols for drug overdoses are contributing to more morbidity and mortality in this population by instilling fear and distrust in the system, and need to be improved.

In terms of the larger structural and environmental forces that may impact the high utilization of emergency services in this population, respondents did report barriers to accessing general medical care including lack of transportation, long wait times to see providers, and the cost of care. Infrastructural and workforce challenges, particularly in rural areas, have been well-documented in prior work, and can be addressed through expanding and diversifying delivery care models such as community health workers, pharmacy-based care, and telehealth, as well as optimizing existing provider capacity through peer network supports such as the Extension for Community Healthcare Outcomes (ECHO) program and hub-and-spoke programs for medication for opioid disorder (MOUD) treatment expansion (Komaromy et al., 2016, 2018; Speyer et al., 2018; du Toit et al., 2019; Rawson et al., 2019; Darfler et al., 2020).

Our study has several limitations. Given the lack of racial diversity of our participants and the limited geographic area, these findings may not be transferable to all people who use drugs in this or other rural regions. As the perspectives of people who use drugs are understudied in regard to healthcare services, we believe a small number of in-depth, qualitative interviews were warranted to explore basic themes and guide future research. Notably, we do not explore the perspectives of medical providers in this study, which may limit our interpretations and suggestions for interventions. Many of participants' experiences with inappropriate treatment and some of their interactions with law enforcement may have had legitimate medical or legal standing. However, regardless of the clinical or legal reasoning, these interactions left participants feeling dehumanized and fearful and affected their future medical decisions.

## Conclusion

Drug overdoses, mental health concerns, and skin and soft tissue infections are some of the common conditions that bring people who use drugs into healthcare settings. These medical complications are often stigmatized as being self-inflicted due to their connection to illicit drug use, yet they provide an important opportunity for intervention, referral, and to establish care that can benefit the individual, their families and the communities in which they reside. Breaches of trust threaten those opportunities, which could lead to more serious health consequences including disease outbreaks and deaths. People who use and inject drugs face daily obstacles and discrimination in many facets of their lives. Rather than acting as a reprieve from stigmatizing interactions, healthcare systems often reinforce them. Our study illuminates the perspectives and experiences of people who use drugs when they engage medical services. Further research is recommended to inform interventions with the potential to improve clinical services and overall health outcomes for people who use drugs.

## Data Availability Statement

The datasets presented in this article are not readily available because analytical requests for the data must be submitted to the University of Washington Rural Opioid Initiative Data Coordinating Center. Requests to access the datasets should be directed to Mai Tuyet Pho, mpho@bsd.uchicago.edu.

## Ethics Statement

The studies involving human participants were reviewed and approved by University of Chicago Biological Sciences Division Institutional Review Board. The patients/participants provided their written informed consent to participate in this study.

## Author Contributions

KE, SW, MP, LO, JE, and SF contributed to the conception and design of the study. KE and LO conducted the study interviews. KE and MP coded the study interviews. JE collected the study data and performed the demographic analysis. KR contributed to data analysis and preparation of the manuscript. KE wrote the first draft of the manuscript. All authors contributed to manuscript revision and read and approved the submitted version.

## Conflict of Interest

The authors declare that the research was conducted in the absence of any commercial or financial relationships that could be construed as a potential conflict of interest.
